# Prospects and feasibility of synergistic therapy with radiotherapy, immunotherapy, and DNA methyltransferase inhibitors in non-small cell lung cancer

**DOI:** 10.3389/fimmu.2023.1122352

**Published:** 2023-02-17

**Authors:** Chen Jie, Rumeng Li, Yajie Cheng, Zhihao Wang, Qiuji Wu, Conghua Xie

**Affiliations:** ^1^ Department of Radiation and Medical Oncology, Zhongnan Hospital of Wuhan University, Wuhan, China; ^2^ Hubei Key Laboratory of Tumor Biological Behaviors, Zhongnan Hospital of Wuhan University, Wuhan, China; ^3^ Hubei Cancer Clinical Study Center, Zhongnan Hospital of Wuhan University, Wuhan, China

**Keywords:** DMNTis, immunotherapy, radiotherapy, synergistic effect, NSCLC

## Abstract

The morbidity and mortality of lung cancer are increasing, seriously threatening human health and life. Non-small cell lung cancer (NSCLC) has an insidious onset and is not easy to be diagnosed in its early stage. Distant metastasis often occurs and the prognosis is poor. Radiotherapy (RT) combined with immunotherapy, especially with immune checkpoint inhibitors (ICIs), has become the focus of research in NSCLC. The efficacy of immunoradiotherapy (iRT) is promising, but further optimization is necessary. DNA methylation has been involved in immune escape and radioresistance, and becomes a game changer in iRT. In this review, we focused on the regulation of DNA methylation on ICIs treatment resistance and radioresistance in NSCLC and elucidated the potential synergistic effects of DNA methyltransferases inhibitors (DNMTis) with iRT. Taken together, we outlined evidence suggesting that a combination of DNMTis, RT, and immunotherapy could be a promising treatment strategy to improve NSCLC outcomes.

## 1 Introduction

As the most common cause of cancer-associated death mortalities, NSCLC accounts for 80-85% of lung cancers (1). There has been continuous progress in the treatment of NSCLC in recent years, but the 5-year overall survival rate (OS) is about 25%, calling for further improvement (2). In recent years, immunotherapy, most notably ICIs, has made remarkable breakthroughs in the field of NSCLC, bringing long-term survival benefits to a proportion of NSCLC patients. Nevertheless, most patients do not achieve satisfactory treatment efficacy due to primary or acquired treatment resistance (3). The treatment of NSCLC relies heavily on RT. In the past decades, with the emergence of intensity-modulated radiotherapy (IMRT), stereotactic body radiotherapy (SBRT), image-guided radiotherapy (IGRT) and other modes, the precision of RT has become increasingly high. Thus, the local control rate is much higher with better spare of adjacent normal tissue and organs. Although there have been a few cases of patients experiencing an abscopal effect with RT, the efficacy of RT in controlling systemic lesions remains limited (4, 5).

Given the respective limitations of ICIs and RT, promising results from the combination of these therapies in NSCLC have been reported in a growing number of studies, with synergistic effect by combined ICIs and RT significantly improving patients’ outcome. ICIs blocked inhibitory immune checkpoints such as PD-1, PD-L1, and CTLA-4 to reboot the cancer-immunity cycle (CIC) and prevent tumor immune evasion, thus restoring and maintaining anti-tumor immunity. RT plays a vital role in anti-tumor immunity together with ICIs during CIC. The mechanisms by which RT enhances the anti-tumor immune response are versatile. Firstly, RT can promote the release of neoantigens and the expression of MHC-I molecules, thereby activating antigen-presenting cells such as dendritic cells (DCs) (6, 7). Secondly, RT can regulate the TME, effectively promote the infiltration of CD8+ T lymphocytes by increasing the levels of chemokines CXCL10 and CXCL16 in TME, and reducing immune-related suppressor cells in the tumor stroma, shifting the TME transition from cold tumor to the hot tumor (8, 9). Thirdly, RT can enhance anti-tumor immunity through activating cGAS/STING and IFN-I pathways, the activation of which is supposed to significantly rely on RT dose and fractionation (2, 10). Lastly, RT increases the expression of PD-L1 on cancer cells, thus enhancing the therapeutic effect of PD-L1 antibodies (11, 12). On the other hand, ICIs can not only activate killer T cells, but also normalize tumor vessels, reduce hypoxia, and increase tumor sensitivity to RT (13, 14).

The synergistic mechanism of RT and ICIs has laid a solid foundation for their combined application in clinical practice. Currently, numerous phase 3 clinical trials are being launched or have been completed for various stages of NSCLC, as summarized in **Table 1**. The landmark PACIFIC phase 3 trial demonstrated that the addition of a PD-L1 inhibitor following concurrent chemoradiotherapy (cCRT) brought about clinical effects in patients with locally advanced NSCLC (15). In patients with early-stage operable NSCLC, a recent phase 2 randomized trial of SBRT in combination with durvalumab versus durvalumab alone showed statistically significant major pathological response rates of 53.3% (95% confidence interval 34.3% - 71.7%) and 6.7% (95% confidence interval 0.8% - 22.1%), respectively (16). A pooled analysis combining PEMBRO-RT and MDACC trials in metastatic NSCLC revealed that compared with pembrolizumab alone, pembrolizumab plus RT significantly improves median progression-free survival (mPFS) and median overall survival (mOS) (17). Although existing clinical trials have shown promising therapeutic effects from early to advanced stages of NSCLC, current mode of immunoradiotherapy (iRT) still has obvious limitations. Less than 50% of patients diagnosed with locally advanced NSCLC survive long-term (18). The objective response rate (ORR) of the PEMBRO-RT trial did not meet the study’s pre-defined endpoint criteria for meaningful clinical benefit in advanced NSCLC patients receiving Pembrolizumab after SBRT (19). Although current studies on the synergistic mechanism of combination of immunotherapy and RT have accumulated certain evidence, the activation of RT on tumor immunity still exists only by chance. Several patients have RT resistance and ICIs resistance, resulting in poor response to iRT or short duration of immunotherapy maintenance after RT. The efficiency of iRT remains to be improved. Besides, the optimal drugs combination with RT, the sequence of RT and immunotherapy, and the RT dose pattern remain unclear. To date, there is no optimal biomarker to guide clinicians in selecting advantaged populations. In addition, the incidence of abscopal effect of RT is very low in clinical application, and the mechanism research needs to be further in-depth.

**Table 1:** Summary of ongoing or completed phase 3 trials of immunotherapy combined with RT for NSCLC.

NCT number	Study name	No. of patients	Inventions	Setting	Status
Early-stage NSCLC					
NCT03924869	MK-3475-867/KEYNOTE-867	530	Pembolizumab vs Placebo	Consolidation treatment after SBRT	Recruiting
NCT03833154	PACIFIC-4	733	Durvalumab vs Placebo	Consolidation treatment after SBRT	Recruiting
NCT04214262		480	Atezolizumab vs Placebo	Induction/Consolidation treatment combined with SBRT	Recruiting
Locally-advanced NSCLC					
NCT03519971	PACIFIC-2	328	Durvalumab vs Placebo	Consolidation treatment after concurrent chemoradiotherapy	Completed
NCT04597671	NVALT28	170	Durvalumab+PCI vs Durvalumab+observation	Consolidation treatment plus PCI after CRT	Recruiting
NCT04092283		660	Concurrent CRT and Durvalumab vs CRT	Concurrent immunotherapy	Recruiting
NCT04380636	MK-7339-012/KEYLYNK-012	870	Pembrolizumab+Olaparib vs Pembolizumab+Placebo	Consolidation treatment after CRT	Recruiting
NCT04026412	CheckMate73L	888	Nivolumab+Ipilimumab vs Durvalumab	Consolidation treatment after CRT	Active, not recruiting
NCT04513925	SKYSCRAPER-03	800	Atezolizumab+Tiragolumab vs Durvalumab	Consolidation treatment after CRT	Recruiting
NCT03728556	GEMSTONE-301	381	Sugemalimab vs Placebo	Consolidation treatment after CRT	Completed
Advanced NSCLC					
NCT03774732	NIRVANA-LUNG	460	Pembrolizumab+ChT+3D-CRT/SABR vs Pembrolizumab+ChT	First-line treatment	Recruiting
NCT03391869	LONESTAR	360	Nivolumab+Ipilimumab+LCT vs Nivolumab+Ipilimumab	First-line treatment	Recruiting
NCT03867175		112	Pembrolizumab+SBRT vs Pembrolizumab	Consolidation treatment after First-line systemic treatment	Recruiting

NSCLC, non-small cell lung cancer; RT, radiotherapy; CRT, chemoradiotherapy; SBRT, stereotactic body radiotherapy; PCI, prophylactic cranial irradiation; ChT, chemotherapy; SABR, stereotactic ablative radiotherapy; 3D-CRT, three dimensional conformal radiotherapy; LCT, local consolidation therapy.

DNA methylation is the most common and best studied epigenetic modification that has been involved in immune evasion and radioresistance in numerous studies. Emerging evidence suggested that targeting DNA methylation could be a promising strategy to substantially enhance the effect of iRT. In this review, we focus on the regulation of DNA methylation on ICIs resistance and radioresistance in NSCLC and elucidate the potential synergistic effects of DNA methylase inhibitors (DNMTis) in patients treated with iRT. The combination of DNMTis, RT, and immunotherapy is worthy of further study to improve NSCLC outcomes.

## 2 DNA methylation

**Figure 1:**
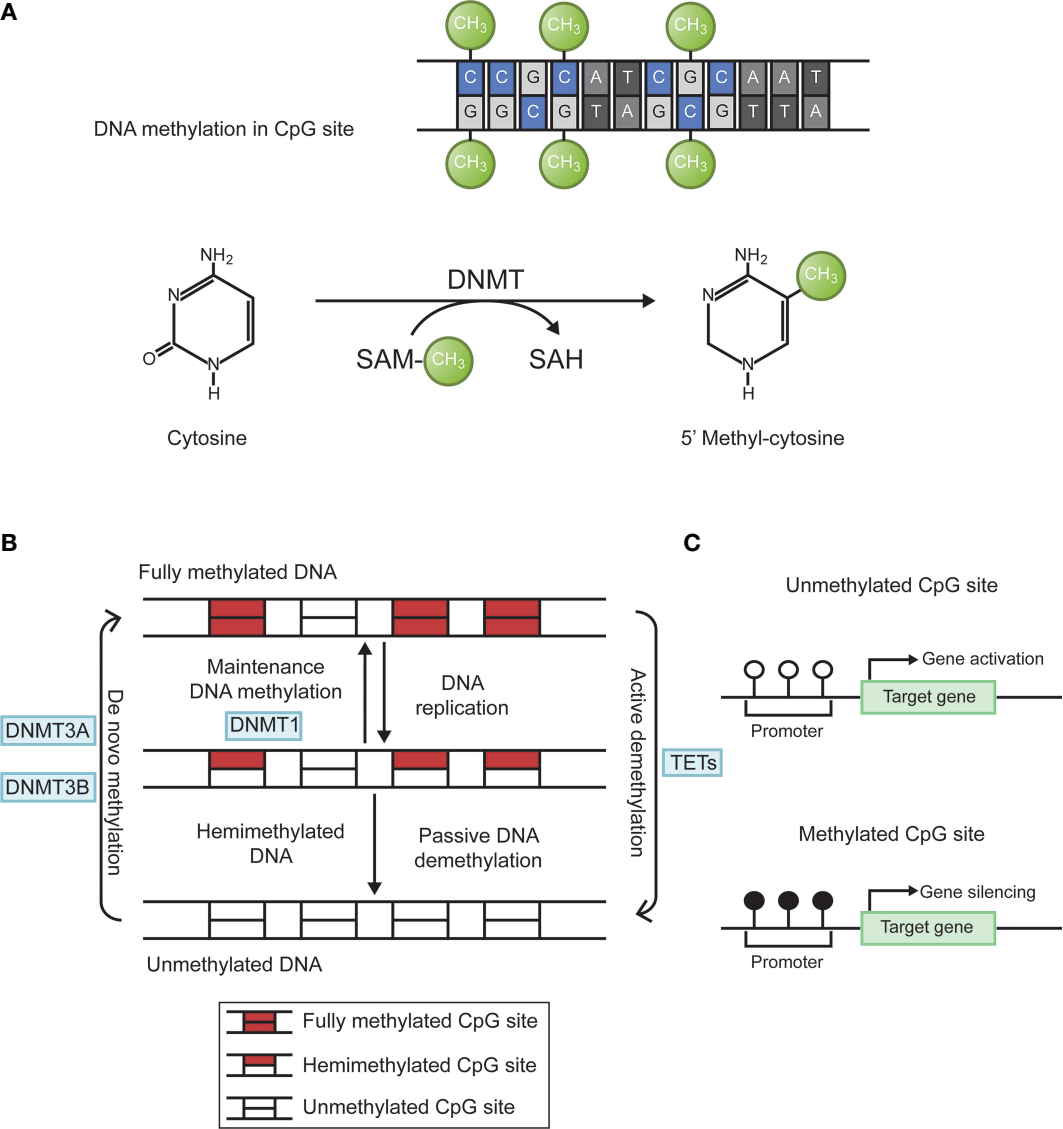
Biological process of DNA methylation. **(A)** Cytosine is catalyzed by DNMTs to form 5 ‘methyl-cytosine, with SAM as the methyl donor. **(B)** DNA methylation is dynamically regulated by DNMTs and TETs. Figure 1-B adapted from Jeltsch (26). **(C)** Hypermethylation of target gene promoter causes gene silencing. DNMTs, DNA methyltransferases; SAM, S-adenosyl methionine; CpG, cytosine-guanine; TETs, ten-eleven translocation enzymes.

Epigenetics is the study of heritable changes in gene expression without affecting a gene’s primary nucleotide sequence. The term now refers to inherited changes in gene expression that do not result from altered DNA sequences. It includes DNA methylation, histone modification, chromosome remodeling, and RNA regulation. Abnormal DNA methylation is the most studied form of epigenetic modification (20, 21). The main forms of DNA methylation are 5-methylcytosine (5-mC); 5-hydroxymethylcytosine (5-hmC); n6-methyladenine (N6-mA); n4-methylcytosine (4mC); 7-methylguanine (7-mG). In eukaryotes, methylation mainly occurs in cytosine. Under the action of DNA methyltransferases (DNMT), methyl is added to the fifth carbon position of cytosine to produce 5-methylcytosine (22, 23). When methylated, gene shows stronger inertia *in vitro*. For example, sodium bisulfite can convert unmethylated cytosine into uracil, but cannot change cytosine within methylated cytosine guanine (CpG) islands. the living state showed a decrease in gene expression activity. Through this modification, DNA conformation, DNA stability, DNA-protein interaction, and chromatin structure will change, thereby controlling gene expression (24). DNA methylation reaction is divided into two types. *De novo* methylation occurs in both DNA strands, whereas maintenance methylation takes place in one DNA strands and leave the other strand unmethylated. DNA methylation is mainly catalyzed by specific enzymes named DNA methyl transferases (DNMTs). There are four main DNMTs: DNMT1, DNMT3A, DNMT3B, and DNMT3L. After DNA replication is completed, DNMT1 is the most important enzyme in the methylation reaction that catalyzes the transfer of methyl groups to newly synthesized DNA strands, a phenomenon known as maintenance methylation. DNMT3A and DNMT3B are responsible for catalyzing the reaction of new methylation sites on the nucleic acid chain, called *de novo* methylation. DNMT3L is a regulatory enzyme in the DNA methyltransferase family without methyltransferase activity, and its main role is to regulate the activity of other methyltransferases (25). In addition, DNA methylation status at each CpG site is dynamically regulated by the local activity of DNA demethylation enzymes (e.g., TET enzymes) and DNA replication rate (26). The biological process of DNA methylation is shown in **Figure 1**.

## 3 DNA methylation and NSCLC

In the 5’untranslated region (5’UTR) of the promoter region and the first exon region of the gene, the CpG sequence density is high, more than 5 times the average levels. These regions are characterized by repeated guanine and cytosine-rich fragments called CpG islands and have typical lengths of 200 bp ~ 1 kb. As an important epigenetic event in the occurrence and development of cancer, methylation of CpG islands plays a key role in gene silencing (27). Because the local hypermethylation of CpG islands occurs earlier than the malignant transformation and proliferation of cells, methylation can be used for cancer screen, prevention, and early diagnosis. It is common for CpG islands to be unmethylated in normal cells. However, in tumor cells, DNA methylation occurs in key regulatory regions associated with tumor related genes, which is supposed to be a major contributor to tumor transformation (28).

### 3.1 Connection of DNA methylation and the progression of NSCLC

**Figure 2:**
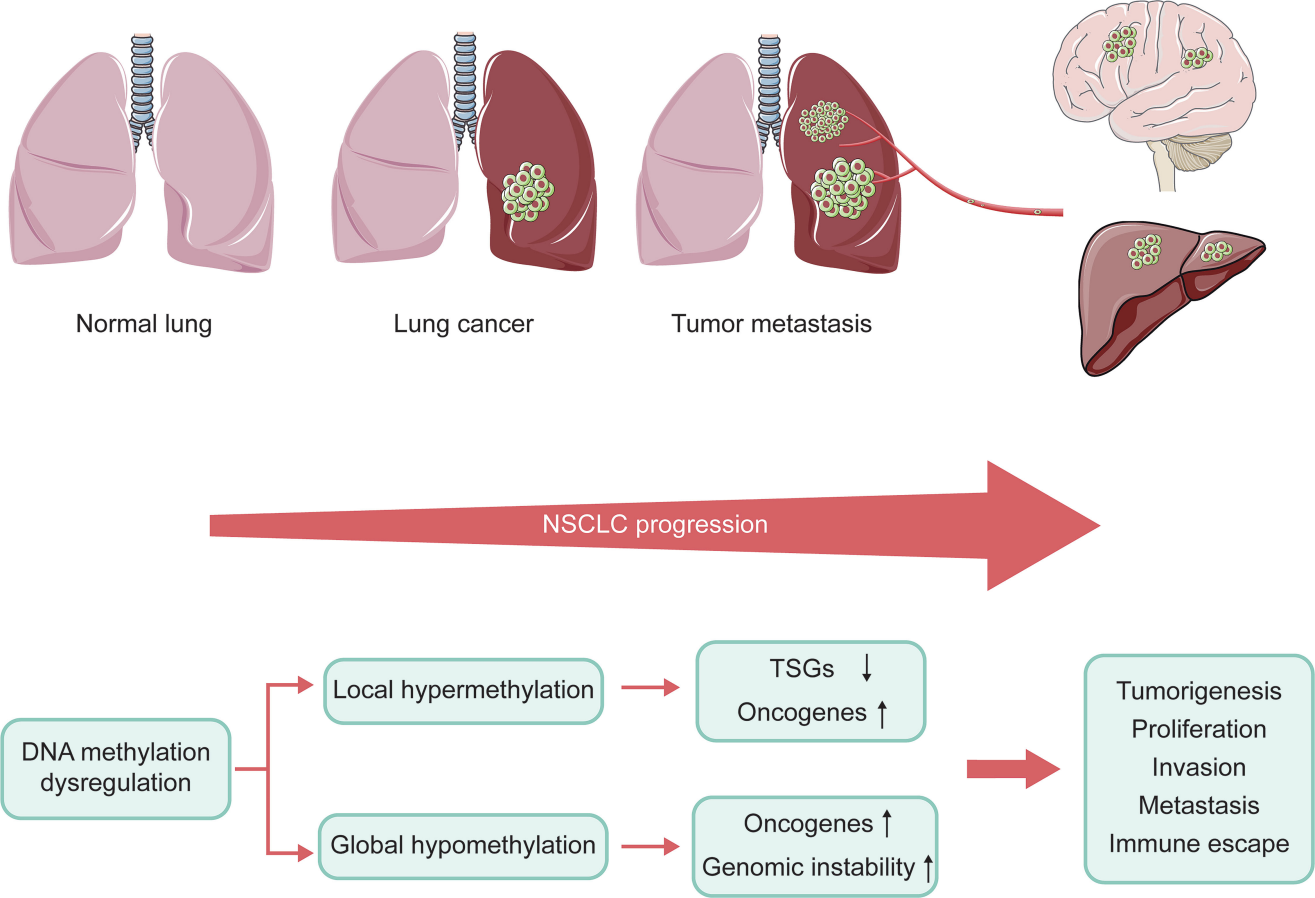
Connection of DNA methylation and the progression of NSCLC. NSCLC progression is caused by inactivation of TSGs, oncogenes activation, and genomic instability due to dysregulation of DNA methylation. NSCLC, non-small cell lung cancer; TSGs, tumor suppressor genes.

DNA methylation plays a vital role in the progression of NSCLC. During the development of cancer, numerous genes are silenced or activated through epigenetic modification (29). Many tumors exhibit abnormal DNA hypomethylation at the whole genome and hypermethylation at specific promoter sites, even at early stages of the disease (30). The genome-wide DNA hypomethylation leads to activation of oncogenes and retrotransposon elements and makes the genome instable. Daskalos and his colleagues revealed that the increase of hypomethylation of retrotransposon elements (LINE-1 and Alu) caused enhancement of transcription, closely related to increased genomic instability observed in NSCLC (31). Consistent with this, enhanced genome-wide hypomethylation is also relevant to higher mutation, copy number variation, allele imbalance burden and Treg/CD8 ratio in the progression of lung cancer (32). However, hypermethylation of local promoter region mainly causes the inactivation of tumor suppressor genes (TSGs). Despite differences in detection methods and samples, a large number of studies consistently reported CpG islands (CGIs) hypermethylation of various TSGs in NSCLC (33). These TSGs play an important role in DNA repair, apoptosis as well as cell cycle regulation, which are significantly associated with tumor progression. In preclinical study, these genes were shown to be reactivated by DNMTis, further confirming that they were silenced by DNA hypermethylation (34). Perhaps paradoxically, DNA hypermethylation has also been shown to induce the activation of tumor-promoting genes. Hypermethylation of *telomerase reverse transcriptase* (*TERT*) gene promoters has been shown to enhance *TERT* expression in most tumor types (35). However, the mechanism of hypermethylation leading to increased gene expression remains unclear. In summary, the inactivation of TSGs, activation of oncogenes, and genomic instability caused by DNA methylation dysregulation result in tumorigenesis, proliferation, invasion, metastasis, as well as immune escape **(** **Figure 2** **)**.

### 3.2 DNA methylation and early diagnosis of NSCLC

Widschwendter et al. highlighted the importance of DNA methylation detection for tumor risk screening, providing new opportunities for cancer patients (36). Studies have shown that DNA methylation was an early event in the occurrence and development of lung cancer (37). In lung cancer, genes with methylated specific CpG islands included *CDKN2A, RASSF1A, RARbeta, MGMT, GSTP1, CDH13, APC, DAPK, TIMP3*, etc (38, 39). Shivapurkar et al. proposed the use of methylation detection for early diagnosis and screening of lung cancer after finding abnormal methylation status of *APC, CDKN2A/p16, HS3ST2*, and *RASSF1A* genes in the sputum of lung cancer patients (40). Hypermethylated status of promoters was detected in the blood, bronchial lavage, induced sputum, and even pleural effusion of primary NSCLC patients (41–43). Burbee et al. explored the hypothesis that *RASSF1* encoded a tumor suppressor gene in lung and breast cancer, and found that *RASSF1A* was a potential tumor suppressor gene in lung and breast cancer. Epigenetic inactivation occurs through hypermethylation of its promoter region, resulting in a worse prognosis (44). Another study showed that 3 of the 5 subjects with *RASSF1A* gene methylation were diagnosed with lung cancer about 1 year after sampling (45). At present, in NSCLC, the gene with more methylation variation is *CDKN2A*. Belinsky and colleagues explored the methylation level of the *CDKN2A* gene or *MGMT* gene promoter in the body fluids of patients 3 years before clinical diagnosis of NSCLC (37). Another study showed that among the 8 subjects with *CDKN2A* gene methylation, 3 subjects were diagnosed with lung cancer about 1 year after sampling (46). Other studies have demonstrated that abnormal methylation status of the *SHOX2* gene helps to distinguish lung tumor tissues from normal tissues (46–48). Testing for DNA methylation status could help effectively screen patients in the early stages of lung cancer.

### 3.3 DNA methylation and prognosis and metastasis of NSCLC

Based on TCGA cancer multi-omics data analysis, Liu and colleagues found that transcription factor and DNA methylation site coupling can regulate gene expression dynamics, thus affecting the prognosis of patients (49). Marsit et al. found that *FANCF* promoter methylation was an important predictor of poor survival in lung adenocarcinoma (LUAD), and that *FANCF* methylation was related to the age of smoking and drinking (50). The study by Liu et al. found that *TMEM196* hypermethylation effectively distinguishes lung cancer patients from normal subjects, and those with *TMEM196* hypermethylation had a worse prognosis (51). Wrage et al. found that adenocarcinoma patients with *HERC5* promoter hypermethylation in NSCLC had worse survival, and *HERC5* promoter hypermethylation was significantly associated with brain metastasis (52). According to Yu et al., LUAD patients with hypomethylated *FAM83A* had a poorer prognosis with high levels of *FAM83A* expression (53). Previous researchers have found that in patients with NSCLC, the higher the methylation level of *EPHB6*, *HS3ST2*, *DAL-1*, and *TMEM88* genes, the greater the risk of metastasis, while the lower methylation of the *ELMO3* gene, the greater the risk of NSCLC metastasis. These studies have shown that the status of methylation of certain genes may be related to cancer metastasis (54–58).

The prognosis of brain metastasis in patients with NSCLC is very poor. Xu et al. detected differences in methylation sites in different groups, and for the first time demonstrated that it may be possible to predict the risk of lung cancer brain metastasis based on DNA methylation (59). Bacha et al. found that patients with NSCLC whose *MGMT* promoter region was methylated after brain surgery had a poorer survival time than patients whose *MGMT* was not methylated (60). These two studies provide a new idea for predicting the risk of brain metastasis of NSCLC. In conclusion, detection of methylation level may provide a new approach to predicting a patient’s prognosis based on a specific gene. We summarize the connection between some common DNA methylation genes and the prognosis and metastasis of NSCLC **(** **Table 2** **)**.

**Table 2:** Association of DNA methylation genes with prognosis and metastasis in non-small cell lung cancer.

Gene	DNA methylation level	Result	Reference
EPHB6	Increased	more prone to metastasis	(54)
FANCF	Increased	Poorer prognosis	(50)
HS3ST2	Increased	More prone to metastasis	(55)
TMEM88	Increased	More prone to metastasis	(57)
DAL-1	Increased	More prone to metastasis	(56)
TMEM196	Increased	Poorer prognosis	(51)
HERC5	Increased	Poorer prognosis	(52)
ELMO3	Decreased	More prone to metastasis	(58)
RASSF1A	Increased	Poorer prognosis	(61)
FAM83A	Decreased	Poorer prognosis	(53)
MGMT	Increased	More prone to metastasis	(60)

## 4 DNA methylation leads to immunotherapy resistance in NSCLC

DNA methylation dysregulation may alter cell phenotypes, reshape TME, as well as influence the status of certain signaling pathways and antigen presentation. These factors allow tumor cells to evade immune surveillance, leading to immunotherapy resistance. DNMTis are expected to reverse these conditions, which could further enhance the effect of immunotherapy.

### 4.1 Mechanisms of immunotherapy resistance

Anticancer immunotherapy, especially ICIs, is changing the treatment paradigm in numerous cancer types, and has been widely used in clinical practice with remarkable clinical benefits (62–64). Although a few patients have achieved significantly longer survival, most patients would have treatment failure due to primary or acquired treatment resistance (65, 66). From tumor-specific antigens recognition to cross-presentation, from T cell activation to recruitment, ICIs treatment resistance occurs at every step of the tumor immune cycle (67). The causes of ICIs treatment resistance include tumor-intrinsic and tumor-extrinsic mechanisms.

#### 4.1.1 Tumor-intrinsic mechanism

The tumor-intrinsic mechanism mainly refers to low tumor mutation burden (TMB) and expression of PD-L1, loss of neoantigen expression, deficiency of antigen presentation, activation of driver genes, and dysfunction of specific pathways in tumor cells. These changes in tumor cells can lead to the development of immune resistance.

##### 4.1.1.1 TMB

TMB is a critical biomarker, which can serve as a predictor of the efficacy of ICIs. Yarchoan et al. demonstrated that a significant positive correlation could be observed between TMB and objective response rate (ORR) with ICIs in 27 tumor types (68). Based on the follow-up study of KEYNOTE-158, patients with high TMB had better ORR compared to those with low TMB who received pembrolizumab (29% vs 6%, p<0.05) (69). Compared with high TMB cancer types (e.g. NSCLC and melanoma), and low TMB cancer types such as pancreatic cancer and prostate cancer show poorer response to the treatment effect of ICIs (70). Mechanistically, increased TMB might boost the expression of tumor antigens and improve the efficacy of immunotherapy (71).

##### 4.1.1.2 PD-L1

In addition to TMB, PD-L1 is another valid predictor, which is widely utilized in clinical practice. Although numerous studies have demonstrated that high expression of PD-L1 in tumor cells can mediate immune escape, current clinical trials have proved that high expression of PD-L1 can make tumor cells more sensitive to ICIs (72–75). A derivative study of KEYNOTE-001 demonstrated that advanced NSCLC patients with high PD-L1 expression (>= 50% of tumor cells) had better ORR than those with low PD-L1 (< 50% of tumor cells) expression who received pembrolizumab (75). Compared to platinum-based chemotherapy group, pembrolizumab group prolongs PFS and OS for stage IV NSCLC patients with higher PD-L1 expression (>=50% of tumor cells) (74). Similar to low TMB, low PD-L1 expression does not indicate an absolutely poor immunotherapy response, and different functions of PD-L1 may exist rather than absolute inhibition or promotion (72, 73, 76–78). The low TMB and expression of PD-L1 prior to ICIs initiation are associated with the primary resistance of tumor. The dynamic changes of PD-L1 expression status and TMB during treatment may be related to the acquired resistance of tumor. Despite that high TMB and PD-L1 expression play a critical part in predicting treatment efficacy to ICIs, the forecast of ICIs treatment efficacy is far more than TMB and PD-L1 evaluation.

##### 4.1.1.3 Tumor neoantigens

In the process of tumorigenesis, cancer cells will undergo genetic alterations to promote the production of neoantigens. Neoantigens are recognized by both innate immune cells and primed adaptive immune cells that cooperate to destroy newly formed cancer cells (79). Loss of neoantigens is considered to be one of the causes of ICIs resistance which may interfere with the recognition and presentation of antigens by immune cells. George and colleagues analyzed the genomic distinctions between the primary tumor and metastasis of a case of metastatic uterine leiomyosarcoma after ICIs resistance. They found that, in genomics, the uniquely harbored biallelic PTEN gene of metastasis resistant to treatment was lost, and the expression of two neoantigens was reduced. These two neoantigens showed strong immune reactivity to the patient’s T cells *in vitro*, indicating a lasting immune memory (80). A large cohort study by Anagnostou and colleagues found that 7 to 18 neoantigens in whole-exome sequencing of tumors disappeared after patients with NSCLC developed ICIs resistance (81). Anaplastic lymphoma kinase (ALK) fusion can be detected in 3%-8% of patients with NSCLC. Patients with ALK fusion had decreased expression of neoantigens and increased amounts of immunosuppressive cells through PI3K-AKT and MEK-ERK pathways, causing poor effects of single-agent immunotherapy (82–84). Tumor cells mainly regulate the loss of neoantigens by reducing the expression of neoantigens-related genes and the loss of mutant alleles (85).

##### 4.1.1.4 Antigen presentation

Besides the deprivation of neoantigens, the decline of antigen presentation ability can also lead to ICIs resistance. Major histocompatibility complex class I (MHC I) is a vital member in the immune process, involving in antigen processing and presentation. The engagement of MHC I molecule on the surface of cancer cells and the T cell receptor (TCR) on the surface of CD8+T cells promotes the activation of CD8+T cells (86). Beta-2 microglobulin (B2M) is one of the vital components that makes up the heavy chain of MHC I, playing a role in stabilizing MHC I (87). An American study confirmed that in NSCLC, *B2M* gene mutation could cause the deletion of MHC I molecule on the cancer cell surface, further lead to the recognition obstacle of CD8+ T cells, and induce immune resistance (88). NSCLC patients with epidermal growth factor receptor (EGFR) gene mutation have poor responses to ICIs (82). When the EGFR pathway is activated, the signal transducer and activator of the transcription 3 (STAT3) is upregulated, as a downstream molecule of the EGFR pathway, which leads to the decrease of MCH I expression (89). In addition, immune cell dysfunction and gene loss associated with antigen presentation may also contribute to ICIs resistance (90–92).

##### 4.1.1.5 Other signal pathway

The abnormality of the special signal pathway can also cause immunotherapy resistance. The absence of an IFNγ signal protects tumor cells from recognition and attack by immune cells. IFNγ is produced by tumor-specific T cells and performs an effective anti-tumor immune response by recognizing corresponding receptors on cancer cells or antigen-presenting cells. Moreover, IFNγ has the ability to boost tumor antigen presentation by increasing MHC I expression. At the same time, it can directly repress the proliferation of cancer cells, promote apoptosis, and recruit immune cells to cause anti-tumor effects (91, 93). Thus, mutations and deletions of IFNγ pathway-related proteins on tumor cells, such as IFNγ receptors and receptor chains, resulting in resistance to immunotherapy, which is a key factor of primary and acquired immune resistance (94–96). Continuous activation of Wnt (Wingless-type MMTV integration site family)/β-catenin signaling pathway by stabilizing β-catenin eliminates T cells from the TME, causing a “non-T-cell inflamed” TME resistant to ICIs (97). Besides, Wnt/β-catenin signaling could directly suppress the activation of T cells (98).

#### 4.1.2 Tumor-extrinsic mechanism

The tumor-extrinsic mechanism is mainly caused by the change in the host immune microenvironment, which refers to the reduction of cellular components and cytokines related to immune activation in TME and the increase of cellular components and cytokines related to immune suppression in TME. TME is not only the internal environment for tumor cells to survive and develop but also the “main battlefield” for immune cells to kill tumor cells. As a result of low infiltration levels and exhaustion of immune effector cells, immune suppression occurs, thereby mediating immune escape (99). According to the distribution frequency of CD8+T cells, the immune phenotype is divided into three types: the inflamed phenotype, the immune-desert phenotype, and the immune–excluded phenotype. In contrast with the inflamed phenotype, the latter two phenotypes seldom have a response to ICIs, leading to primary resistance (100). T cell exhaustion is considered to be a dysfunctional status caused by immunosuppressive TME and chronic/persistent presence of tumor antigens (101). Continuous antigens stimulation brings about T cell exhaustion, and CD8+T cell exhaustion is supposed to be a vital reason for tumor immune resistance (102). Other inhibitory immune checkpoints can also enhance the immune suppression function by promoting T cell exhaustion (103). In addition, Immunosuppressive cells such as myeloid-derived suppressor cells (MDSCs) and tumor-associated macrophages (TAMs) have the ability to promote tumor immune escape (104, 105). The regulatory T cells (Tregs) can directly contact or secrete immunosuppressive cytokines such as IL-10, IL-35, and TGF-β to inhibit the function of effector T cells (106). Increasing the secretion of cytokines such as indole-2, and 3-dioxygenase (IDO) can inhibit immune response (107). Metabolic changes in the TME can also lower immune effects by releasing product of metabolism to repress immune cell infiltration (108). Once the delicate balance between the “gas pedal” and the “brake” in the TME is broken and develop along the direction of negative regulation, it will lead to immune resistance. Similarly, we can utilize these mechanisms to enhance antitumor immunity by promoting positive regulation.

### 4.2 DNMTis promote anti-tumor immunity

The concept of immunotherapy has brought new progress in the treatment of cancer. However, cancer cells and host immune cells are prone to immune tolerance after the interaction. In the context of cancer, epigenetics can reshape the TME, promote tumor growth, and escape the immune system. For example, aberrant DNA methylation can provide survival benefits to tumor cells by silencing genes necessary for antitumor activity. DNMTis can reduce DNA hypermethylation in CG-rich regions (CpG islands) of tumor suppressor gene promoters and restore transcriptional activity at these sites, thereby altering the immune response (109, 110). Zhang et al. analyzed the expression level of PD-L1 in patients with NSCLC after chemotherapy, immunotherapy, and EGFR-TKI treatment. This study demonstrated that immunotherapy inhibited the expression of PD-L1 through promoter hypermethylation, while PD-L1 increased in the other two treatments. A xenograft NSCLC model was used to clarify the relationship between anti-PD-1 treatment and PD-L1 promoter. The study found that the methylation level of PD-L1 was significantly decreased in AZA-treated tumor cells, and the PD-L1 mRNA level was enhanced (111). To their delight, tumor volume was significantly decreased in patients treated with methylation inhibitors combined with immunotherapy. Therefore, this combination may be a promising way to eliminate ICIs resistance.

By summarizing previous studies, we believe that epigenetic therapy affects tumor immune resistance through the following factors. First, DNMTis enhance antigen presentation by increasing the expression level of MHC I molecule and tumor antigens such as tumor-testis antigens (CTAs) and endogenous retroviruses (ERVs) (112, 113). After ERVs promoter demethylation, it can induce viral mimicry or activate inhibited retroviruses to express double-stranded RNA, thereby recruiting more cytotoxic T lymphocytes into the TME and changing the production of cytokines, thereby eliminating tumor cells (114–117). Second, DNMTis can activate TLR3 and MDA5 after up-regulating dsRNA, thereby activating the classic type I interferon signaling pathways (117). After activation of the type I interferon signaling pathway, the composition of the TME and the expression of MHC I molecule on the cell surface can be regulated by increasing the percentage of CD8+T cells and natural killer (NK) cells in the TME and reducing the percentage of macrophages and bone marrow-derived suppressor cells (118, 119). Third, epigenetic silencing of T helper factor 1 (TH1) chemokines is a new mechanism of tumor immune escape. Peng et al. showed that DNMT1 and EZH2 inhibitors can re-activate the production of TH1 chemokines, boost the infiltration of effector T cells, inhibit tumor progression, and enhance the efficacy of ICIs (120). Fourth, effector cells in TME during such a long-term stimulation will lead to cell effector function gradually being suppressed, in such a situation, T cells are prone to exhaust. The ‘ tired ‘ state of immune cells often leads to an integral part of immune tolerance and evasion (121, 122). Ghoneim et al. found that *de novo* DNA methylation acquired after CD8 + T cell exhaustion can lead to further exhaustion of T cells, while DNMTis can reduce CD8 + T cell exhaustion by inhibiting DNMT3a-mediated *de novo* DNA methylation (123). The activation and differentiation of CD8 + T cells are the results of stimulation by professional antigen-presenting cells after antigen presentation. Epigenetic mechanisms play an key part in determining the fate of T cells (124). For example, epigenetic therapy can inhibit MYC activity to enhance type I interferon signaling and induce CCL5 production, and then CCL5 can bind to CCR5 to recruit more CD8 + T cells (119). Further, DNMTis can remarkably increase IFN-γ-induced Cxcr3 chemokine (Cxcl9/10/11) expression, promoted Th1 polarization, and helped CD8 + T cells enhance cytotoxic activity and enhance anti-PD-1 antibody response (125, 126). Finally, DNA demethylating agents can not only change the immune response through a variety of ways but also reprogram cancer cells (127, 128), making tumors more sensitive to checkpoint inhibition (117). These suggest that DNMTis play a vital role in anti-tumor immunity.

### 4.3 Clinical application of DNMTis

DNMTis have the ability to inhibit methylation formation in the CpG region (129). The use of low doses can reverse gene silencing, while high doses can exert cytotoxicity in killing tumor cells (130). DNMTis have been proved to have good curative effect in the field of hematologic tumors and are widely used in clinical practice. In solid tumors, a growing number of single-agent and combination therapy studies have been completed or are ongoing, promising to provide helpful insights into antitumor therapy.

#### 4.3.1 Currently available DNMTis

The landmark drugs of clinically approved epigenetic therapy are azacitidine (AZA) and 5-2′-deoxycytidine (decitabine), two nucleoside classes of DNMTis that were discovered by researchers in the 1960s (131). After several decades of development, these two drugs can be used to treat a variety of hematologic tumors, including myelodysplastic syndrome (MDS), acute myeloid leukemia (AML), and become the new standard of non-intensive first-line treatment (132–134). Despite their significant efficacy in hematological tumors, the toxicity, low response rate, and poor chemical stability limit their use in the treatment of solid tumors (135, 136).

In the past few decades, some novel nucleoside DNMT inhibitors and non-nucleoside DNMT inhibitors have been identified and used as antitumor drugs. We summarize the commonly used DNMTis in **Table 3**.

**Table 3:** Summary of DNA methyltransferase inhibitors.

Substance Group	Drug Name	Target
Nucleoside analogs	Decitabine (5-aza-2’-deoxycytidine, Dacogen R, DAC)	DNMT1 and DNMT3A
	Azacitidine (5-azacytidine, Vidaza R)	DNMT
	5-Fluoro-2′-deoxycytidine (FdCyd)	DNMT1
	CC-486	DNMT
	Guadecitabine (SGI-110)	DNMT
	4′-thio-2′-deoxycytidine (TdCyd)	DNMT1
	Sinefungin	DNMT
	Zebularine	DNMT1, CDA
Non-nucleoside analogs	Nanaomycin A	DNMT3B
	MG98	DNMT1
	1-Hydrazinophthalazine	DNMT
	CBC12	DNMT
	Epigallocatechin gallate (EGCG)	DNMT
	Procainamide	DNMT1
	Psammaplin A	DNMT, HDAC
	RG 108	DNMT1
	SGI-1027	DNMT1, 3A and 3B
	Thioguanine	DNMT
	MC3343	DNMT1
	MC3353	DNMT1
	BIX-01294	DNMT3A, DNMT1 and G9a

DNMT, DNA methyltransferase; CDA, cytidine deaminase; HDAC, histone deacetylase.

Recently, a phase 1 clinical trial showed that CC-486 (an oral azacitidine) as monotherapy has a good therapeutic effect on advanced nasopharyngeal carcinoma. 37.5% of patients had partial response (PR), and 50% of patients achieved stable disease (SD) (137). CC-486 combined with immunotherapy deserves further study. Guadecitabine (SGI-110) is a second generation of DNMTis, which solves the disadvantage that the first-generation DNMTis are easy to be deaminated by cytidine deaminase (CDA), and improves the chemical stability (138). Zebularine is another nucleoside analog. Unlike azacitidine and decitabine, zebularine is very stable in neutral aqueous solutions and less toxic (139). Psammaplins are a class of phenolic compounds isolated from marine sponges, which can inhibit DNMT and histone deacetylase (HDAC) (140). Arce et al. proposed that hydralazine may act directly on DNMT by embedding the three-dimensional catalytic group pocket of DNMT, and the mechanism of its demethylation needs further study (141). MG98 is a non-nucleoside analogues directly acting on the 3 ‘ end of DNMT1 mRNA. In phase 1 clinical trial, 33 patients with advanced solid malignancies received MG98 treatment. One patient achieved PR and another remained stable. Inhibition of DNMT1 expression was observed in 26 patients (142).

#### 4.3.2 DNMTis monotherapy in NSCLC

At present, the commonly used DNMTis are azacitidine (5 ‘-azacytidine) and decitabine (5-aza-2 ‘ -deoxycytidine), which are cytidine analogs. DNMTis have a wide range of cellular effects, through phosphorylation and DNA binding, inducing DNA hypomethylation, apoptosis, or activation of specific genes, such as tumor suppressor genes, to exert anti-tumor effects (143). Momparler et al. conducted a clinical study on the toxicity and clinical efficacy of decitabine in patients with advanced NSCLC. The study included 15 patients with NSCLC, and one patient survived more than 81 months (144). The delayed mode of action of decitabine was proposed by Montparell et al., which provides a new strategy for treating NSCLC. A phase 2 study to assess the efficacy and safety of 5-fluoro-2 ‘ -deoxycytidine in combination with tetrahydrouridine was initiated by the National Cancer Institute in 2009. This study included 95 patients with NSCLC, breast cancer and other cancers (https://clinicaltrials.gov/ct2/show/NCT00978250). The PFS of 25 NSCLC patients was 2.3 months. Severe AEs occurred in 38 of the 93 patients, the most common of which was gastrointestinal reactions. Schiffmann et al. included 10 patients with refractory advanced NSCLC in a phase 1/2 trial to evaluate the tolerance of azacitidine combined with atenolol. The treatment was well tolerated and an objective response was observed. The median survival time was 6.4 months (145).

#### 4.3.3 DNMTis combined with immunotherapy in NSCLC

In previous studies, when NSCLC patients only received single-agent methylation inhibitors, such as DNMTi azacytidine, only 4% of patients showed an objective response, and the effect was not ideal (145). Wrangle showed that DNA hypomethylation agent azacytidine (AZA) could increase the level of PD-L1 expression in cell lines (146). In addition, Chiappinelli et al. proposed several possible signal transduction mechanisms for how epigenetic therapy can boost the efficacy of immunotherapy, which provides evidence for clinical trials of NSCLC (147). Therefore, we believe that the combination of epigenetic therapy and immunotherapy may produce a synergistic anti-tumor efficacy. Here we list some clinical trials of DNMTis combined with immunotherapy in patients with NSCLC **(** **Table 4** **)**.

**Table 4:** Clinical trials of DNMTis combined with immunotherapy in NSCLC.

Inhibitor target	Inhibitor (name)	Study type	Status	Clinical trial number
**DNMT + PD-1 + CDA**	Decitabine + Nivolumab +THU	Phase 2	Completed	NCT02664181
**DNMT + HDAC + PD-1/PD-L1**	Azacitidine + Entinostat + ICIs	Subsequent study of a phase 2 trial	Completed	(146)
**DNMT + PD-1**	CC-486 + Pembrolizumab	Phase 2	Active, not recruiting	NCT02546986
**DNMT + PD-1**	Guadecitabine + Pembrolizumab	Phase 1	Active, not recruiting	NCT02998567
**DNMT + HDAC + PD-1**	Azacitidine + Entinostat + Nivolumab	Phase 2	Active, not recruiting	NCT01928576
**DNMT + HDAC + PD-1**	Guadecitabine + Pembrolizumab + Mocetinostat	Phase 1	Active, not recruiting	NCT03220477
**DNMT + PD-1 + CDA**	Decitabine + Pembrolizumab + THU	Phase1/2	Recruiting	NCT03233724
**DNMT + CTLA-4 + PD-1**	Guadecitabine + Ipilimumab + Nivolumab	Phase 2	Not recruiting	NCT04250246
**DNMT + PD-1**	Decitabine + Camrelizumab	Case report	Completed	(148)
**DNMT + IDO-1 + PD-1**	Azacitidine + Epacadostat + Pembrolizumab	Phase1/2	Completed	NCT02959437

DNMT, DNA methyltransferases; CDA, cytidine deaminase; THU, tetrahydrouridine; ICIs, immune checkpoint inhibitors; IDO-1, indoleamine 2,3-dioxygenase; HDAC, histone deacetylase; CTLA-4, cytotoxic T lymphocyte-associated antigen.

In a follow-up study of combined epigenetic therapy for advanced treatment-refractory NSCLC, six patients received ICIs, three of whom achieved PR and two achieved SD, all of which lasted more than 8 months (146). The good results obtained in this study demonstrated the potential of DNMTis combined with immunotherapy and opened the prelude to a series of subsequent clinical trials. In a case report, Han et al. reported the unexpectedly good results of 3 patients with advanced NSCLC carrying adverse ICI biomarkers, such as low TMB. Surprisingly, all three patients responded well to low-dose DAC combined with camelizumab, with slight AEs, indicating that low-dose DAC can sensitize ICIs (148). A dose-escalation study was conducted on guadecitabine, a second-generation DNA methylation inhibitor, to determine its effect on solid cancers. Among the 12 assessable NSCLC patients, 10 patients had previously received ICIs, of which 5 (42%) had been in disease control for more than 2 years (149). In a phase 2 trial, 100 patients who had previously received platinum-based therapy were given either pembrolizumab plus oral AZA or a placebo, but PFS was not improved (150). A large phase 2 study was initiated in 2013, which included 101 patients with advanced NSCLC who were treated with azacitidine and antidote in combination with nivolumab or nivolumab alone. The results of this clinical trial have not been released (https://clinicaltrials.gov/ct2/show/NCT01928576). In 2016, 13 NSCLC evaluation studies were recruited to evaluate whether the drug tetrahydrouridine-decitabine (THU-Dec) combined with nivolumab is more effective than nivolumab alone in the treatment of NSCLC patients (https://clinicaltrials.gov/ct2/show/NCT02664181). Among them, 8 were in an experimental group and 5 were in the control group. The latest results in August 2022 showed that 4 of the 8 patients in the experimental group developed progression, 2 were SD, and 2 were PR; among the 5 patients in the control group, 1 progressed, 3 were stable, and 1 was partially relieved. The PFS was 69 and 227 days, respectively. As of the latest results, the OS for the experimental and control groups was 389.5 days and 844 days, respectively. In the same year, a 1/2 phase study (https://clinicaltrials.gov/ct2/show/NCT02959437) was conducted in USA, UK, and Spain to evaluate the safety and tolerance of azacitidine combined with pembrolizumab and epalrestat. The study included 70 subjects with advanced solid tumors and previous stage IIIB or IV NSCLC and stage IV microsatellite-stabilized colorectal cancer. The study was permanently discontinued in February 2019 and there were no patients in treatment groups B and C. Treatment group A was divided into two groups according to different doses, 62 patients with 100 mg INC B24360 and 8 patients with 300 mg INC B24360. These results demonstrated that the all-cause mortality rate of the 100 mg INCB24360 group was 66.13%, and the risk of serious adverse events was 45.16%. The all-cause mortality of patients with 300 mg INCB24360 was 50%. The risk of serious AEs (adverse events) was 37.5%. In 2017, 34 castration-resistant prostatic cancer and NSCLC were included to evaluate the safety and toxicity of decitabine (SGI-110) combined with pembrolizumab (MK3475) in patients with refractory solid tumors (https://clinicaltrials.gov/ct2/show/NCT02998567). In the same year, 28 NSCLC patients were included to assess the safety and dose selection of a 1/1b phase study (https://clinicaltrials.gov/ct2/show/NCT03220477) in the treatment of patients with advanced NSCLC with bortezomib combined with guadicitabine and moxitinib. The clinical trial was first published in 2017 and is expected to be completed in July 2023. The trial results have not yet been released. Overall, it is safe to use DNMTis. Epigenetic therapy does not directly affect cell cycle progression or apoptosis, but rather regulate some genes to cause global changes in cellular transcriptional programs, and therefore do not have an immediate cytotoxic effect. But there is much more to learn in how to make better use of DNMTis in clinical settings, such as a more detailed understanding of drug resistance mechanisms and more clinical trials.

## 5 DNA methylation leads to radioresistance in NSCLC

RT is a vital treatment for NSCLC, however, radioresistance is considered to be the main cause of the poor effect of RT for NSCLC. The underlying mechanism of radioresistance remains to be clarified. To improve the radiosensitization, investigators concentrate mainly on ameliorating the hypoxia state, increasing DNA damage, and affecting the cell cycle. At present, the commonly used radiosensitizers in clinical practice are 5-fluorouracil, platinum, and gemcitabine (151–153). Nevertheless, these drugs not only have limited radiosensitizing effects but also increase the toxicity of RT (154). Now, accumulating evidence has shown that aberrant epigenetic alterations are related to the radioresistance of NSCLC. Abnormal DNA methylation of radiosensitivity related genes in promoter region leads to radioresistance. DNA methylation has attracted the most interest among epigenetic modifications in radioresistance. Drugs regulating methylation-related genes have great clinical potential for improving the radiosensitivity of NSCLC.

### 5.1 DNA methylation regulates radiosensitivity in NSCLC

#### 5.1.1 Mechanism of DNA methylation regulating radiosensitivity

DNA methylation is combined with histone modification, resulting in RNA polymerase binding to this region, causing the silence of related genes, especially tumor-suppressive genes (155, 156). It is believed that DNA methylation is linked to a variety of cellular events, such as apoptosis, the progression of the cell cycle, the regulation of mitotic checkpoints, and repairing DNA damage. These cellular regulatory actions can affect radiosensitivity (157, 158). The clinical radiobiological effect is based on five radiobiological factors, including DNA repair, repopulation, re-oxygenation, redistribution, and radiosensitivity, which determine the rate of tumor response to radiotherapy. The first four of these factors are regarded as 4 ‘R’, which are regulated by DNA methylation-related genes and influence radiosensitivity (154, 159). Here, we summarize genes involved in radiosensitivity regulated by DNA methylation **(** **Table 5** **)**.

**Table 5:** Radiosensitivity related genes regulated by DNA methylation in NSCLC.

Gene	Methylation site	NSCLC Cell line	Methylation status	Response to RT	Gene function
PTEN	Promoter	H1299	Hypermethylation	Resistant	Suppressing DNA damage repair, re-oxygenation
MicroRNA-9 gene	Promoter	A549	Hypermethylation	Resistant	Suppressing DNA damage repair
OTUD4	Promoter	A549, H460	Hypomethylation	Sensitive	Suppressing DNA damage repair
SERPINB5	Promoter	H1299	Hypermethylation	Resistant	Suppressing tumor cells proliferation
TM4SF4	Promoter + 5′-UTR	A549, Calu-3	Hypomethylation	Resistant	Promoting tumor cells proliferation
IGFBP-3	Promoter	Sample from patients	Hypomethylation	Resistant	Promoting tumor cells proliferation
S100A6	Promoter	H1299	Hypermethylation	Resistant	Modulating cell cycle
Dab2	Promoter	LK2	Hypermethylation	Sensitive	Inhibition of Wnt pathway

NSCLC, non-small cell lung cancer; RT, radiotherapy; 5′-UTR, 5’ untranslated region.

#### 5.1.2 DNA methylation and re-oxygenation related genes

One of the important reasons for tumor resistance to RT is the lack of oxygen in the tumor due to abnormal or dysfunctional blood vessels. This hypoxic state results in less DNA damage for the same dose of radiation (160). Furthermore, hypoxia drives resistance to RT through the accumulation and stabilization of *HIF-1 α* (161). *HIF-1 α* is one of the re-oxygenation-related genes, and its high expression is significantly related to radioresistance. The hypoxic state of cells will reduce radiosensitivity (162). Wang et al. found that overexpression of miR-320a caused an increase in the radiosensitivity of NSCLC cells by facilitating methylation of *PTEN via HIF-1α*/*KDM5B* axis inhibition (163). Therefore, we speculate that hypermethylation status may affect NSCLC radiosensitivity through to silence of re-oxygenation-related genes.

*PTEN*, a robust tumor suppressor gene, which is often inactivated in numerous various types of cancer cells. *PTEN* can lose its function through DNA methylation in NSCLC (164). Meyn and colleagues found that transferring the wild-type *PTEN* gene into an H1299 NSCLC cell line with a known methylated *PTEN* promoter would enhance its sensitivity to irradiation. The repair of DSBs induced by irradiation was suppressed in H1299 cells pretreated with adenoviral-mediated *PTEN* (165).

#### 5.1.3 DNA methylation and DNA repair-related genes

Unlike re-oxygenation-related genes, for DNA repair inhibitor genes, demethylation can improve the radiosensitivity of tumor cells. A preclinical study revealed that DNMTis, such as 5-aza-2’-deoxycytidine, and zebularine are promising drugs to enhance radiosensitivity in NSCLC, most possibly *via* regulating the damage of the DNA repair process (158). Among the radiation-responsive genes, activating nuclear factor-kappa B1 (NFκB1) promotes DNA damage repair and cell survival. According to the report, microRNA-9 can sensitize H1299 cells to ionizing radiation by inhibition of NF-κB1 (166). Jin and colleagues found that microRNA-9 increased radiosensitivity in NSCLC and this effect is remarkably affected *via* its promoter methylation status. This study revealed that over-expression of DNMT1 significantly decreased the expression of microRNA-9 by up-regulating the methylation level of its promoter. Furthermore, the promoter methylation level of microRNA-9 was significantly enhanced in response to irradiation (167). Thus, we believe that DNMT1 inhibitors can enhance microRNA-9 expression by down-regulating its promoter methylation level, resulting in increasing the radiosensitivity of NSCLC. *OTUD4* has been supposed to participate in DNA damage repair pathways containing GG-NER and the alkylation damage repair pathway (168, 169). Mi and colleagues showed that *OTUD4* is inactivated in promoter methylation status and its down-regulation is related to inferior prognosis in NSCLC. Overexpression of *OTUD4* impairs DNA double-strand breaks (DSBs) homologous recombination (HR) repair, augments cell cycle arrest, and increases cell death induced by ionizing radiation (IR). *OTUD4* could be a potential target for radiosensitizing NSCLC. Furthermore, a study indicates that *OTUD4* radiosensitizes NSCLC *via* ATM/CHK2/P53 signaling and suppresses homology-directed repair of DNA DSBs induced by IR (170).

#### 5.1.4 DNA methylation and cell proliferation-related genes

DNA methylation level of cell proliferation-related genes correlates with the radiosensitivity in NSCLC. The *SERPINB5* gene is a tumor suppressor gene, which plays an important part in suppressing the ability of cancer cell proliferation, metastasis, and invasion (171). A study by Kim et al. found that the *SERPINB5* gene was hypermethylated in radioresistant H1299 cells. PCR (polymerase chain reaction) revealed higher expression of SERPINB5 in radiosensitive lung cancer cells than radioresistant cells (172). The transmembrane 4L six family member 4 (*TM4SF4*) protein is a cell surface glycoprotein that has the function of modulating cell proliferation (173). A study in South Korea demonstrates that *TM4SF4* overexpression in lung cancer cells leads to radioresistance *via* IGF1-induced IGF1R activation. The detection of the CpG island methylation level of the *TM4SF4* gene revealed that lung cancer cells with hypo-methylated status resulted in the overexpression of *TM4SF4* (174). Insulin-like growth factor-binding protein-3 (IGFBP-3) serves as a vital role in cell proliferation, growth and survival by mediating the PI3 kinase (PI3K)/Akt pathway (175). A study in Spain showed that the hypomethylated *IGFBP-3* promoter correlates with radioresistance in NSCLC. The study revealed that patients with promoter hypomethylation status did not benefit from adjuvant RT after R0 surgery (176).

#### 5.1.5 DNA methylation and cell cycles regulation related genes

The radiosensitivity of cancer cells in different cell cycles is different. Compared with cells in the M phase and G2 phase, cells in the S phase are generally less sensitive to RT. Tumor cells in the M phase and G2 phase are selectively killed, which leads to the phenomenon of redistribution (177). Some studies have proved that genes driving cell cycles to G2/M with hypermethylation status could lead radioresistance in different tumor cell lines (154). The *S100A6* gene functions as modulating cell cycle. In radioresistant cell lines of NSCLC, the promoter of this gene is hypermethylated, while in radiosensitive cell lines, the promoter is hypomethylated (172).

#### 5.1.6 DNA methylation and CSCs related genes

There is increasing evidence showing that some radioresistant cancer cells have the characteristics of stem cells, which are known as cancer stem cells (CSCs) (178–180). A study has shown that CSCs markers can be modulated by methylation. DNA methylation is related to the Wnt pathway involved in the activation of CSCs (181, 182). Classic Wnt signaling pathway is crucial in NSCLC. In resected samples and cell lines of NSCLC, hypermethylation decreases Wnt inhibitor level, which correlates with a poor prognosis. Wnt signaling may also enhance radioresistance, and the inhibitors of Wnt may restore radiosensitivity (183). Wang et al. compared the hypermethylated lung cancer with the hypomethylated lung cancer of the *Dab2* gene promoter, they found radiation remarkably suppresses proliferation and invasion of NSCLC with hypermethylated promoter but is less efficient in NSCLC with hypomethylated promoter. This study demonstrates that the methylation level of the Dab2 gene promoter might have the promise to predict the radiosensitivity of NSCLC. Moreover, these results indicate that ionizing radiation could induce demethylation of lung cancer cells with hypermethylation of the Dab2 gene promoter *via* down-regulating DNMTs and MeCP2, then increasing the Dab2 expression, which may enhance radiosensitivity. The process may inhibit the Wnt pathway (184)..

### 5.2 DNMTis combined with radiotherapy

Although numerous preclinical studies have demonstrated the significant radiosensitization effect of DNMTis, there are no result related to DNMTis combined with radiotherapy for NSCLC. The clinical trials such as NCT03445858, NCT01707004, and NCT04174196, that are currently being recruited or have been completed, focusing on lymphoma and hematological neoplasms, have shown the promise of DNMTis combined with RT. Therefore, increasing clinical trials are needed to undertake to explore the potential benefit of DNMTis combined with RT.

## 6 Feasibility of immunoradiotherapy combined with DNMTis

**Figure 3:**
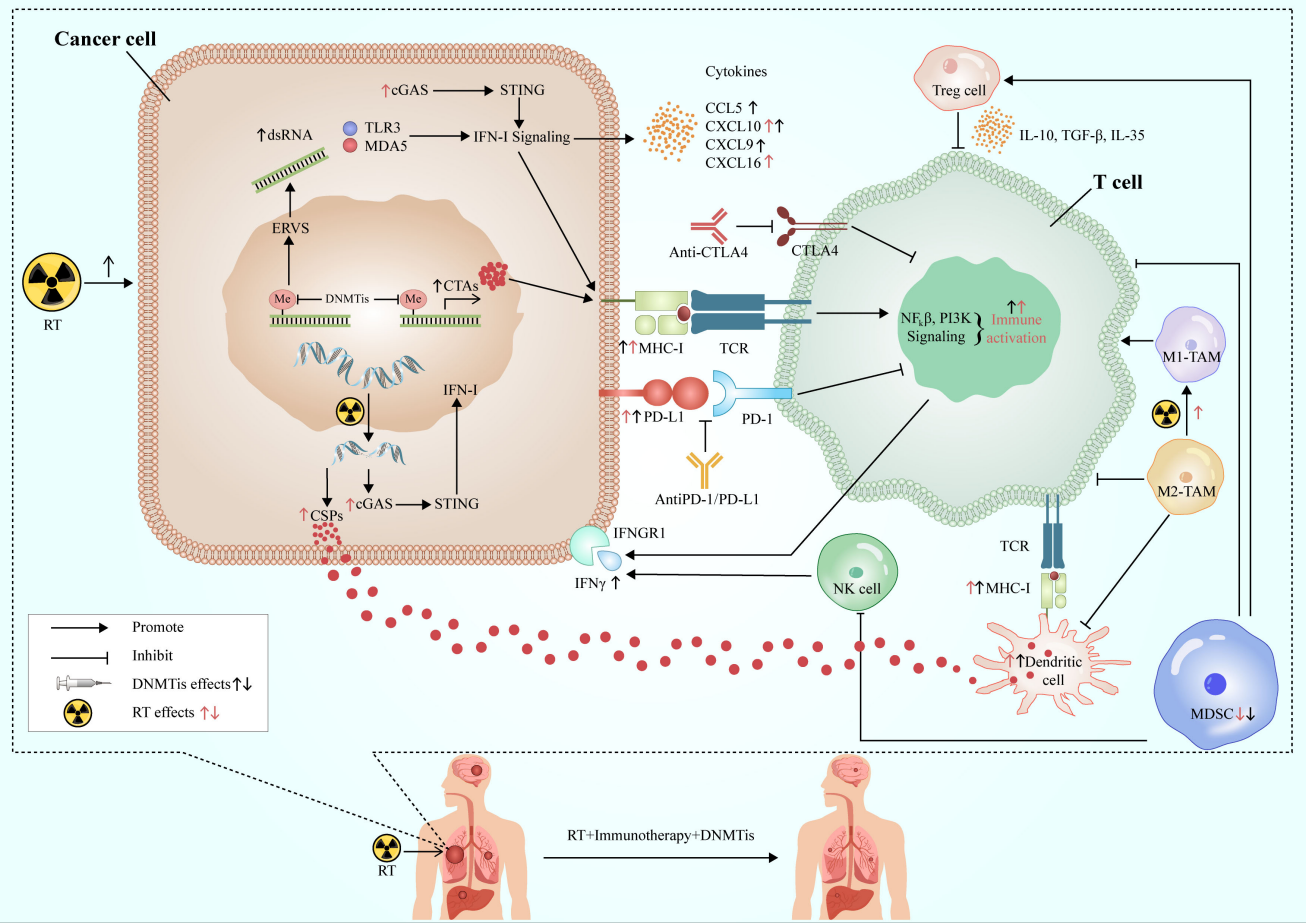
Synergistic effects of RT, immunotherapy and DNMTis. RT acts on cancer cells to produce double-stranded DNA, release cancer-specific peptides, and activate the cGAS-STING-IFN-I pathway to promote anti-tumor immunity. DNMTis remove methylation from endogenous retroviruses and activates type I interferon signal *via* TLR3 and MDA3 sensors, while releasing cancer testis antigens, which are involved in antigen processing and presentation, and anti-tumor immunity. RT and DMNTis can also promote the expression of PD-L1 in cancer cells and improve the efficacy of immunotherapy. In addition, they can also affect the TME, which is conducive to the formation of anti-tumor immune microenvironment. DNMTis make cancer cells more sensitive to RT and immunotherapy, which work in concert with each other to achieve long-lasting and effective anti-tumor effects. RT, radiotherapy; CSPs, cancer-specific peptides; ERVs, endogenous retroviruses; CTAs, cancer testis antigens; DNMTis, DNA methyltransferase inhibitors; MDSC, myeloid-derived suppressor cell; TAM, tumor-associated macrophage; cGAS, cyclic GMP-AMP synthase; STING, stimulator of interferon genes; MHC-I, major histocompatibility antigen-I; IFN-I, interferon I; IFNGR1, interferon gamma receptor 1; TCR, T cell receptor.

RT has a good local control effect on the tumor, but its systemic anti-tumor effect is relatively limited, while immunotherapy is the contrary. RT can promote anti-tumor immunity by increasing neoantigen release and changing TME and other mechanisms. The combination of DNMTis and the former two can not only enhance the sensitivity of RT to increase the ability of tumor local control but also promote the further release of tumor antigens and improve immunogenicity. At the same time, DNMTis can also increase the production of neoantigens and the expression of MHC I molecule, and improve the antigen presentation ability of immune cells. In addition, DNMTis can activate cytotoxic T lymphocytes, inhibit the function of immunosuppressive cells, up-regulate the positive cytokines of immune regulation, create a TME conducive to immunotherapy, and improve the systemic effect of immunotherapy. The three complement each other and play a better and more powerful anti-tumor effect **(** **Figure 3** **)**.

However, the toxicity of DNMTis is a problem that cannot be underestimated. Some DNMTis have been extensively used in clinical practice with controllable AEs, but the AEs of treatment in combination with ICIs have not been widely studied, especially in long-term outcome. With the advent of new DNMTis, the toxicity aspect of the drug is acceptable. In addition, clinical studies associated with low-dose DNMTis and RT as induction before ICIs are also worthy of future exploration. For different stages of NSCLC, in combination with different ICIs, more in-depth exploration of the dose, frequency, time, and sequence of RT, as well as better control of the side effects, can make patients more benefit from combination therapy.

## 7 Challenges and perspectives

DNA methylation is a very stable sign that can be detected in various types of biological samples, including tumor tissue and body fluids (185). At present, more and more studies believe that DNA methylation can be used as a biomarker for early diagnosis of lung cancer, and can be used to assist the pathological classification of lung cancer (186–188). Moreover, DNA methylation is associated with TMB in NSCLC and has potential as a biomarker for immunotherapy, which can also be used to predict the efficacy of chemotherapy and molecular targeted drugs (33, 61, 189). Despite all this, the current studies on DNA methylation mainly focus on single genes or some special gene groups. For patients receiving DNMTis treatment, there is no clear research indicating which specific groups can benefit, which requires more research to find more effective biomarkers. Current advances in gene sequencing technology promise to select specific populations for epigenetic drugs to achieve long-lasting therapeutic effects and tolerable AEs. At present, chemoradiotherapy combined with immunotherapy has achieved good results in NSCLC, but it still has limitations. On this basis, adding DNMTis will hopefully bring more benefits to the survival of patients with NSCLC. DNMTis combined with iRT, a “chemotherapy-free” treatment mode, is expected to achieve better efficacy and lower toxicity than concurrent chemoradiotherapy, which is well worth our exploration and challenge.

## 8 Conclusion

The advent of iRT has been a boon for patients with NSCLC and has proven to be a fruitful research field for NSCLC. Future research will focus more on improving existing iRT. DNMTis are promising therapeutic agents for reversing radiation resistance as well as immune resistance due to their ability to sensitize tumors to radiation and significantly activate tumor immune responses. We eagerly look forward to the emergence of clinical studies of iRT combined with DNMTis, using epigenetic therapy to reverse tumor treatment resistance and sensitize to iRT, benefiting a broader group of patients than currently exists.

## Author contributions

CX provided fund support and designed the conception of the review. CJ, RL, and QW wrote the main manuscript text. CJ and RL were responsible for drawing the figure and designing tables. QW, ZW, and YC critically revised the article. All authors contributed to the article and approved the submitted version.
